# Assessment of Decision-Making and Material Selection for Vital Pulp Therapy in Deep Carious Lesions: A Study at the Faculty of Dentistry, King Abdulaziz University

**DOI:** 10.7759/cureus.47463

**Published:** 2023-10-22

**Authors:** Reem Ajaj, Mona Alsulaiman

**Affiliations:** 1 Department of Restorative Dentistry, Faculty of Dentistry, King Abdulaziz University, Jeddah, SAU; 2 Department of Endodontics, Faculty of Dentistry, King Abdulaziz University, Jeddah, SAU

**Keywords:** reversible pulpitis, pre-operative vitality test, deep carious lesions, pulp capping, root canal treatment, vital pulp therapy

## Abstract

Purpose: The purposes of this study were to assess decision-making, material selection, and management of deep carious lesions in permanent teeth requiring vital pulp therapy (VPT); investigate the intradepartmental and interdepartmental consensus in the management of those cases; and correlate this study's results to the current scientific literature, clinical experience, and postgraduate training among staff and postgraduate students at the Faculty of Dentistry, King Abdulaziz University.

Materials and methods: The survey included faculty from pedodontics, endodontics, and restorative/operative dentistry; postgraduate students; and interns, excluding specific categories such as retired faculty, external trainers, non-faculty hospital specialists, general practitioners, students, interns outside the institution, and other departments. An anonymous electronic questionnaire was developed and validated. Ethical approval was obtained, and the questionnaire was distributed to all 148 English-proficient members of the targeted population via email and WhatsApp, accompanied by a cover letter. The questionnaire encompassed demographic, education, experience, assessment, decision-making, and management sections. Data were collected and analyzed using Microsoft Excel, with results presented using categorical variables, Pareto charts, and statistical tests.

Results: There were 86 responses, representing 58% of the target population, with the key findings including the prominence of "Pre-operative vitality test result" as the most important factor in assessing deep carious lesions, with no significant differences among specialties. The (one-step and one-visit) management approach was preferred by 50% of participants, with no significant specialty differences. For deep carious lesions without pulpal exposure, glass ionomer (GI)/resin-modified glass ionomer (RMGI) base was the top choice, with no variation among all specialties. In cases with pulpal exposure, the one-visit approach (direct pulp capping (DPC), base, and restoration) was the most favored, with no specialty differences. Material availability significantly influenced decision-making, with no specialty variations.

Conclusion: The study highlights the crucial role of pre-operative vitality tests in assessing deep carious lesions for VPT or root canal treatment (RCT). Participants generally favored VPT for cases with normal pulp vitality, with some departmental variation. Controlling bleeding post-pulpal exposure was a central concern. Mineral trioxide aggregate (MTA) was the most commonly used VPT material, followed by Ca(OH)_2_ and Biodentine. Factors such as treatment access, patient compliance, remaining dentin thickness, and oral hygiene had minimal impact on treatment choice. Limited availability of VPT materials was the primary reason for non-use. The survey's acceptable response rate raises concerns about potential non-response bias, though limitations include a lack of data on non-responders. Nevertheless, the survey's strength lies in its comprehensive coverage of key clinical aspects, engaging professionals from diverse specialties and educational levels who are collectively interested in addressing deep caries cases.

## Introduction

Deep carious lesions, characterized by advanced tooth decay penetrating the enamel and dentin, are typically caused by oral bacteria metabolizing sugars and producing acids that erode tooth structure. Inadequate oral hygiene practices, high sugar diets, and factors like reduced saliva flow contribute to their development. Clinically, deep carious lesions can lead to pain, sensitivity, and the risk of pulp infection, which might necessitate dental treatment such as vital pulp therapy (VPT) or root canal treatment (RCT) [1].

VPT is a treatment approach aimed at safeguarding and preserving the vitality, health, and functionality of the dental pulp, which may have been compromised due to dental caries, trauma, or dental procedures. The primary objective of VPT is to facilitate the generation of reparative dentin while ensuring the continued normal function of the affected tooth within the oral cavity. Thus, successfully addressing deep carious lesions through VPT not only alleviates pain and infection but also promotes the long-term health and function of the affected tooth, preventing the need for more invasive procedures like root canal therapy or extraction. The earliest recorded case of VPT is credited to Phillip Pfaff, who applied a layer of gold foil onto an exposed pulp with the intention of promoting pulpal healing [2].

This procedure plays a crucial role in the conservation of affected immature permanent teeth characterized by incomplete root development, where maintaining the integrity of the dental arch is essential during craniofacial growth and development. Later, VPT was expanded to include mature permanent dentition with fully developed roots that exhibit indications of reversible pulpitis. In its guidelines, the American Academy of Pediatric Dentistry (AAPD) states, “Teeth exhibiting provoked pain of short duration relieved with over-the-counter analgesics, by brushing, or upon the removal of the stimulus without signs and symptoms of irreversible pulpitis, have a clinical diagnosis of reversible pulpitis and are candidates for VPT" [3].

However, the notion that VPT should only be considered a viable option in cases where testing results align with the diagnosis of "reversible pulpitis" has recently been challenged [4-6]. Through examination of clinical, biological, and theoretical aspects, the irreversibility of the pulpal disease has been called into question. Histological evidence illustrating the progression of pulpitis indicates the absence of a distinct boundary that would categorize a pulp as beyond repair [5]. Instead, pulpitis can be viewed as a disease exhibiting temporal and spatial gradations, prompting suggestions for the use of terms such as "initial," "moderate," and "severe pulpitis" to describe different gradations of the condition.

Over the years, numerous techniques and materials have been developed for VPT, including indirect and direct pulp capping, pulpotomy, and regenerative endodontics [7]. In a comprehensive study aimed at evaluating the long-term outcomes of VPT, a total of 757 clinical cases that underwent various VPT procedures were monitored to determine success rates, with the longest observation period spanning up to 30 years. Pulp capping materials employed in these cases included chemically pure calcium hydroxide (Ca(OH)_2_), calcium hydroxide-containing bases, mineral trioxide aggregate (MTA), and tricalcium silicate cement. The findings revealed that direct pulp capping achieved a success rate of 73.2%, partial pulpotomy exhibited a success rate of 96.4%, and full pulpotomy demonstrated a success rate of 77.8% for the evaluated cases [5].

Recently, significant progress has been made in understanding pulp biology and harnessing novel dental materials for VPT. Previously employed materials encompassed both biologic and non-biologic substances, which presented alternative treatment options for healthy and partially inflamed pulps [8]. It is acknowledged that the outcomes of VPT can vary, contingent upon factors such as the patient's age, the extent of bacterial contamination, and the degree of pulp inflammation. However, an equally crucial aspect appears to be the selection of the pulp capping material utilized and the subsequent quality of the permanent restoration [9]. Human retrospective studies have reported varying success rates, ranging from 30% to 85% over a period of five to 10 years [10-13]. Appropriate case selection, through a detailed differential diagnosis using multiple tests paired with a careful radiographic interpretation, is paramount for establishing the best treatment for the problem tooth.

In the quest for an optimal material for VPT, a wide array of materials are suggested in the literature to be used as pulp capping protective dressing materials, such as calcium hydroxide, MTA, resin-modified glass ionomer (RMGI), glass ionomer (GI), composite scaffolds, biologically based scaffolds, Bioceramic (Innovative Bioceramix, Vancouver, Canada), and Biodentine (Septodont, Saint-Maur-des-Fosses, France) [14]. Following the release of the American Academy of Endodontics (AAE) position statement on VPT in 2021, a survey was conducted by Wisniewski et al. to understand how U.S. dental schools are educating their students regarding VPT. The survey examined didactic lecture and clinical exposure of undergraduate students in U.S. dental schools to VPT. All schools indicated that students receive a didactic lecture about VPT on permanent teeth. In contrast, 14 of 43 (33%) schools teach VPT on permanent teeth as a technique exercise in the simulation/pre-clinic; 29 of 43 (67%) do not [15]. While previous studies have explored the knowledge and procedures adopted by dentists practicing VPT [16,17], it is worth noting that no prior survey has been conducted in the specific population under investigation here, namely, the Faculty of Dentistry, King Abdulaziz University in Saudi Arabia. Given that material availability can vary between countries due to distinct regulations, country-specific drug administration approvals, and supply availability, it is valuable to conduct research within the present population and make comparisons with findings from other countries.

The objectives of this study were to assess decision-making, material selection, and management of deep carious lesions in permanent teeth requiring VPT; investigate the intradepartmental and interdepartmental consensus in the management of those cases; and correlate this study's results to the current scientific literature, clinical experience, and postgraduate training among restorative, endodontic, and pedodontic staff and postgraduate students and interns at KAUFD.

## Materials and methods

The survey focused on a specific population that includes faculty members and postgraduate (PG) students specializing in pediatric (pedo), endodontics (endo), and restorative/operative (resto) dentistry. It also encompassed interns serving as general practitioners (GPs). Exclusion criteria included retired faculty members, external trainers involved in the internship program, non-faculty specialists in hospitals, GPs, undergraduate students, interns conducting their rotations outside of the Faculty of Dentistry, King Abdulaziz University during the data collection period, and members of other departments at the Faculty of Dentistry, King Abdulaziz University.

An electronic link to the anonymous questionnaire was self-designed and piloted through staff members from the resto and endo departments at the Faculty of Dentistry, King Abdulaziz University who did not participate in the survey. Based on the responses received from the pilot survey, the questions were refined to enhance their validity and reliability. The final questionnaire, as presented in the Appendix, underwent a face validity assessment to ensure the effectiveness of the questions in aligning with the study's objectives. The questionnaire's reliability was verified through a test-retest approach involving the distribution of the same survey twice within a two-week interval to professionals in the field who were not part of the study. Additionally, the content validity of the questionnaire was evaluated by three staff members from the endo, pedo, and resto departments who were not involved in the study. This assessment utilized the scale-level content validity index based on the average method (S-CVI/Ave), and the resulting average index was calculated to be 0.9, indicating strong content validity [18]. Furthermore, the questionnaire underwent a thorough review and received approval from the Faculty of Dentistry, King Abdulaziz University Research Ethics Committee (REC) (Proposal No.: 340-11-21).

The survey questionnaire was circulated through institutional emails, direct WhatsApp messages, and messages to the WhatsApp groups to ensure delivery to all faculty/students within the targeted sample. It was delivered in English only, as all the participants had PG and/or undergraduate English education. To increase compliance, the questionnaire included a cover letter stating that it contained between 19 and 23 questions and would take an estimated four to five minutes to complete. The discrepancy in the number of questions was determined by their relevance to the individual taking the survey. The questionnaire was distributed over a period of six weeks accompanied by weekly reminders.

The purpose of the cover letter was to offer clarification regarding the questionnaire's objective, provide an estimated completion time, and highlight the anonymous nature of the survey. It also provided the option to decline participation and not answer the questionnaire. Additionally, any abbreviations used were clarified in this section. The questionnaire was meticulously crafted to encompass the following sections: demographic information, educational background and experience, assessment, decision-making, and management.

In summary, the survey consisted of four major sections: demographic, education, and experience; assessment; decision-making; and management. The demographic section collected information about participants' age, gender, education, and experience, aiming to explore potential associations between these factors and the strategies employed in managing deep carious lesions. The assessment section examined the factors involved in evaluating deep carious lesions and making treatment decisions, covering patient-related, clinical case-related, and finance-related factors. The decision-making section investigated the factors impacting clinical decision-making processes, material selection, and the consideration of VPT over RCT. The management section focused on treatment options and materials used for deep carious lesions, including approaches for pulp protection and the management of lesions with pulpal exposure. These sections aimed to gather valuable insights into the practices, preferences, and considerations of dental professionals in the assessment, decision-making, and management of deep carious lesions.

The survey link was distributed through the channels mentioned earlier, and responses were gathered in September 2022. The survey was sent to all of the faculty/students with a total number of 148 targeted participants, categorized as follows: endo faculty (n = 40), endo PGs (n = 25), resto faculty (n = 40), resto PGs (n = 10), pedo faculty (n = 10), pedo PGs (n = 15), and interns (n = 8).

Given a population size of 148, a 95% confidence level, and a 5% margin of error, the survey sample size was determined to be 108. The sample size analysis was done using the sample size calculator for surveys from SurveyMonkey at https://www.surveymonkey.com/mp/sample-size-calculator/

All data were collected, tabulated, and subjected to statistical analysis. Microsoft Office Excel was used for data handling and graphical presentation. All variables were categorical variables and expressed as frequency and percentage. For graphical presentation, the Pareto chart was used, which is a bar graph where the bar lengths represent frequency, with the longest bars on the left and the shortest on the right. This chart visually depicts the most commonly chosen factors. A comparison of two proportions was conducted using a z-test. Since the number of individual specialization groups was small, the Fisher exact test was applied for comparing the responses of two groups to a chosen factor. The significance level was considered at P < 0.05 (S), while P < 0.01 was considered highly significant (HS). Two-tailed tests were assumed throughout the analysis for all statistical tests.

## Results

The survey was sent to a total of 148 subjects, representing the total number of the targeted population, of which 40 were endo faculty, 25 endo PGs, 40 resto faculty, 10 resto PGs, 10 pedo faculty, 15 pedo PGs, and eight interns. The total number of responses was N = 86, accounting for 58% of the targeted population. The number and rate of responses for each group were as follows: endo faculty (n = 20, 50%), endo PGs (n = 20, 80%), resto faculty (n = 21, 53%), resto PGs (n = 6, 60%), pedo faculty (n = 6, 60%), pedo PGs (n = 9, 60%), and interns (n = 4, 50%). The findings are presented in both absolute frequencies and percentages.

Demographic data

Out of the total 86 participants, the majority fell within the age group of 30-39 years (32, 37.2%). This was followed by the age group of 23-29 years (26, 30.2%) and 40-49 years (19, 22.1%). The smallest number of participants belonged to the age group above 49 years (9, 10.4%). More than half of the participants were female (54, 62.8%).

In terms of dental training, the participants were distributed across different disciplines in the following order, from highest to lowest percentage: resto faculty (24.4%), endo faculty (23.3%), endo PGs (23.3%), pedo PGs (10.4%), pedo faculty (7%), resto PGs (5.8%), and interns (4.7%). The combined percentage of participants with faculty or postgraduate training is 54.7%. Within the postgraduate training programs, there were varying numbers of participants at different residency levels (R), where R1, R2, and R3 denote the first, second, and third years of residency. Specifically, in the restorative discipline, there were one participant at R1, three participants at R2, and two participants at R3. In the endo discipline, there were nine participants at R1, six participants at R2, and five participants at R3. Lastly, in the pedodontics discipline, there were no participants at R1, two participants at R2, and seven participants at R3. Most faculty members (83%) graduated after 2000, with an equal percentage of faculty graduating in 2000 (8.5%) and before 2000 (8.5%). Regarding postgraduate training, the highest percentage of faculty members (51%) received their training in the USA. This was followed by the UK or European countries (21.2%), Saudi Arabia (15%), and other Arab countries (12.8%).

Among the participants, only a small percentage (11.6%) are classified as academicians and not currently practicing clinically. For those who are practicing, the majority (64.5%) practice in governmental settings, while 31.5% practice in both private and governmental settings, and 4% exclusively practice in private settings.

When considering the frequency of VPT cases per month, the highest reported frequency was one to five cases (71.1%). This was followed by six to 10 cases (13.2%), and a small percentage (9.2%) reported no VPT cases per month. Additionally, a minority (6.5%) reported seeing more than 10 cases of VPT per month. 

Assessment findings

**Figure 1:**
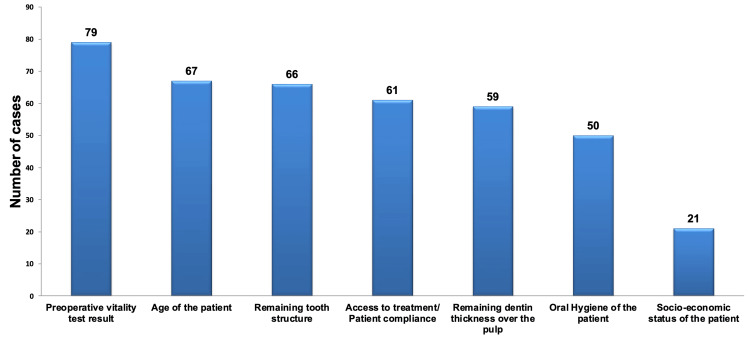
Pareto chart depicting the factors considered when evaluating cases of deep carious lesions for either VPT or RCT, regardless of specialization. The bar graph illustrates that the factor most frequently selected was the pre-operative vitality test result, while the least frequently chosen factor was the patient's socio-economic status. VPT: vital pulp therapy; RCT: root canal treatment.

When analyzing assessment data for deep carious lesions to determine whether VPT or RCT is appropriate before treatment initiation, we observed that the factor most frequently selected, regardless of specialization, was "pre-operative vitality test result." This factor displayed a statistically significant preference over other factors, as illustrated in Figure 1 and detailed in Table 1.

**Table 1:** Multiple comparison z-test results for evaluating the factors relevant to the assessment of deep carious lesions for either VPT or RCT, regardless of specialization. Values denoted by * signify a statistically significant difference (S) with P < 0.05, while ** signifies a high level of statistical significance (HS) with P < 0.01. VPT: vital pulp therapy; RCT: root canal treatment.

		Frequency	Percent	Frequency	Percent	z	P-value	
Pre-operative vitality test result	Age of the patient	79	91.86%	67	77.91%	2.55	0.01064*	P < 0.05 S
Pre-operative vitality test result	Remaining tooth structure	79	91.86%	66	76.74%	2.72	0.00643**	P < 0.01 HS
Pre-operative vitality test result	Access to treatment/patient compliance	79	91.86%	61	70.93%	3.53	0.00042**	P < 0.001 HS
Pre-operative vitality test result	Remaining dentin thickness over the pulp	79	91.86%	59	68.60%	3.83	0.00013**	P < 0.001 HS
Pre-operative vitality test result	Oral hygiene of the patient	79	91.86%	50	58.14%	5.11	0.00000**	P < 0.001 HS
Pre-operative vitality test result	Socio-economic status of the patient	79	91.86%	21	24.42%	8.96	0.00000**	P < 0.001 HS
Age of the patient	Remaining tooth structure	67	77.91%	66	76.74%	0.18	0.85551	P > 0.05 NS
Age of the patient	Access to treatment/patient compliance	67	77.91%	61	70.93%	1.05	0.29439	P > 0.05 NS
Age of the patient	Remaining dentin thickness over the pulp	67	77.91%	59	68.60%	1.38	0.16816	P > 0.05 NS
Age of the patient	Oral hygiene of the patient	67	77.91%	50	58.14%	2.78	0.00545**	P < 0.01 HS
Age of the patient	Socio-economic status of the patient	67	77.91%	21	24.42%	7.02	0.00000**	P < 0.001 HS
Remaining tooth structure	Access to treatment/patient compliance	66	76.74%	61	70.93%	0.87	0.38572	P > 0.05 NS
Remaining tooth structure	Remaining dentin thickness over the pulp	66	76.74%	59	68.60%	1.20	0.23102	P > 0.05 NS
Remaining tooth structure	Oral hygiene of the patient	66	76.74%	50	58.14%	2.60	0.00923**	P < 0.01 HS
Remaining tooth structure	Socio-economic status of the patient	66	76.74%	21	24.42%	6.86	0.00000**	P < 0.001 HS
Access to treatment/patient compliance	Remaining dentin thickness over the pulp	61	70.93%	59	68.60%	0.33	0.73985	P > 0.05 NS
Access to treatment/patient compliance	Oral hygiene of the patient	61	70.93%	50	58.14%	1.75	0.07957	P > 0.05 NS
Access to treatment/patient compliance	Socio-economic status of the patient	61	70.93%	21	24.42%	6.11	0.00000**	P < 0.001 HS
Remaining dentin thickness over the pulp	Oral hygiene of the patient	59	68.60%	50	58.14%	1.42	0.15434	P > 0.05 NS
Remaining dentin thickness over the pulp	Socio-economic status of the patient	59	68.60%	21	24.42%	5.81	0.00000**	P < 0.001 HS
Oral hygiene of the patient	Socio-economic status of the patient	50	58.14%	21	24.42%	4.49	0.00001**	P < 0.001 HS

Across various specialties, there were no statistically significant differences in the selection of assessment factors for the patient's age, remaining dentin thickness over the pulp, pre-operative vitality test result, remaining tooth structure, and factors related to treatment access and patient compliance. However, when it came to the factors of the patient's socio-economic status and oral hygiene, there were statistically significant differences observed among different specialties.

In terms of the socio-economic status factor, a statistically significant difference was found between interns and endo PGs (P < 0.05, determined by Fisher exact probability). Interns tended to prioritize this factor more than endo PGs did, although this difference was not statistically significant with other specialties.

Similarly, regarding the oral hygiene factor, there was a statistically significant difference between the endo faculty and the resto faculty (P < 0.05, determined by Fisher exact probability). The endo faculty tended to consider this factor more important compared to the resto faculty, but this difference was not statistically significant with other specialties. Table 2 presents a breakdown of the number and percentage of each selected factor for either VPT or RCT in cases of deep carious lesions within each specialty.

**Table 2:** The number and percentage (%) of each selected factor for either VPT or RCT in deep carious lesions within each specialty. Resto: restorative dentistry; PG: postgraduate; Endo: endodontics; Pedo: pedodontics; VPT: vital pulp therapy; RCT: root canal treatment.

		Resto faculty (n = 21)	Resto PG (n = 6)	Endo faculty (n = 20)	Endo PG (n = 20)	Pedo faculty (n = 6)	Pedo PG (n = 9)	Intern (n = 4)	Total (N = 86)
Age of the patient	Number	15	6	16	13	6	7	4	67
	%	71.4%	100%	80%	65%	100%	77.8%	100%	77.9%
Socio-economic status of the patient	Number	3	3	7	2	2	1	3	21
	%	14.2%	50%	35%	10%	33.3%	11.1%	75%	24.4%
Oral hygiene of the patient	Number	8	4	15	12	4	4	3	50
	%	38%	66.7%	75%	60%	66.7%	44.4%	75%	58.1%
Remaining dentin thickness over the pulp	Number	17	5	10	13	6	5	3	59
	%	80.9%	83.3%	50%	65%	100%	55.6%	75%	68.6%
Pre-operative vitality test result	Number	20	6	17	19	5	8	4	79
	%	95.2%	100%	85%	95%	83.3%	88.9%	100%	91.9%
Remaining tooth structure	Number	18	4	13	18	5	6	2	66
	%	85.7%	66.7%	65%	90%	83.3%	66.7%	50%	76.7%
Access to treatment/patient compliance	Number	15	4	14	14	5	7	2	61
	%	71.4%	66.7%	70%	70%	83.3%	77.8%	50%	70.9%

Among the participants who considered socio-economic status as a factor (21 participants), the majority practiced in governmental settings (13 participants, 61.9%). The next highest group consisted of those practicing in both governmental and private settings (5 participants, 23.8%). The least represented groups were those who were not practicing (2 participants, 9.5%) and those who were solely private practitioners (one participant, 4.8%).

**Figure 2:**
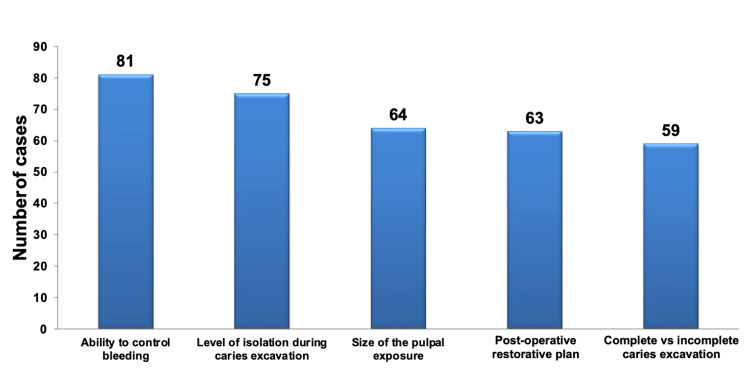
Pareto chart illustrating the factors to consider when evaluating cases of deep carious lesions for VPT or RCT after iatrogenic pulp exposure, regardless of specialization. The bar graph highlights that the most frequently selected factors were the capability to manage bleeding, closely followed by the degree of isolation during caries excavation. VPT: vital pulp therapy; RCT: root canal treatment.

Concerning the factors to evaluate when assessing deep carious lesions for VPT or RCT after accidental pulp exposure, regardless of specialization, the factors deemed most crucial were the ability to manage bleeding and the level of isolation during caries excavation. These two factors exhibited statistically significant importance compared to the other factors, as demonstrated in Figure 2 and detailed in Table 3.

**Table 3:** Multiple comparison z-test results for evaluating the factors relevant to the assessment of deep carious lesions for either VPT or RCT after iatrogenic pulp exposure, regardless of specialization. Values denoted by * signify a statistically significant difference (S) with P < 0.05, while ** signifies a high level of statistical significance (HS) with P < 0.01. VPT: vital pulp therapy; RCT: root canal treatment; NS: not significant.

		Frequency	Percent	Frequency	Percent	z	P-value	
Ability to control bleeding	Level of isolation during caries excavation	81	94.19%	75	87.21%	1.58	0.11525	P > 0.05 NS
Ability to control bleeding	Size of the pulpal exposure	81	94.19%	64	74.42%	3.56	0.00037**	P < 0.001 HS
Ability to control bleeding	Post-operative restorative plan	81	94.19%	63	73.26%	3.72	0.00020**	P < 0.001 HS
Ability to control bleeding	Complete vs. incomplete caries excavation	81	94.19%	59	68.60%	4.31	0.00002**	P < 0.001 HS
Level of isolation during caries excavation	Size of the pulpal exposure	75	87.21%	64	74.42%	2.13	0.03317*	P < 0.05 S
Level of isolation during caries excavation	Post-operative restorative plan	75	87.21%	63	73.26%	2.30	0.02159*	P < 0.05 S
Level of isolation during caries excavation	Complete vs. incomplete caries excavation	75	87.21%	59	68.60%	2.94	0.00328**	P < 0.01 HS
Size of the pulpal exposure	Post-operative restorative plan	64	74.42%	63	73.26%	0.17	0.86227	P > 0.05 NS
Size of the pulpal exposure	Complete vs. incomplete caries excavation	64	74.42%	59	68.60%	0.84	0.39830	P > 0.05 NS
Post-operative restorative plan	Complete vs. incomplete caries excavation	63	73.26%	59	68.60%	0.67	0.50179	P > 0.05 NS

Across various dental specialties, there were no statistically significant differences in the selection of factors related to complete versus incomplete caries excavation, the size of the pulpal exposure, and the ability to control bleeding. However, statistically significant differences were observed among specialties regarding the level of isolation and the post-operative restorative plan.

Concerning the level of isolation, the resto faculty exhibited the highest preference, and this selection was highly statistically significant compared to the pedo faculty (P < 0.001, as determined by Fisher exact probability).

Regarding the post-operative restorative plan, the pedo faculty regarded it as the least important factor in selecting VPT versus RCT, and this difference was statistically significant compared to all other specialties. The resto PGs and interns considered it an important factor in their selection (P < 0.05 with the resto PGs and interns, P < 0.01 with the resto faculty, P < 0.001 with the endo PGs, as determined by Fisher exact probability).

Table 4 provides a breakdown of the number and percentage of each selected factor for either VPT or RCT in cases of deep carious lesions with iatrogenic pulp exposure within each specialty.

**Table 4:** The number and percentage (%) of each selected factor for either VPT or RCT in deep carious lesions with iatrogenic pulp exposure within each specialty. Resto: restorative dentistry; PG: postgraduate; Endo: endodontics; Pedo: pedodontics; VPT: vital pulp therapy; RCT: root canal treatment.

		Resto faculty (n = 21)	Resto PG (n = 6)	Endo faculty (n = 20)	Endo PG (n = 20)	Pedo faculty (n = 6)	Pedo PG (n = 9)	Intern (n = 4)	Total (N = 86)
Level of isolation during caries excavation	Number	21	6	16	18	2	8	4	75
	%	100%	100%	80%	90%	33.3%	88.9%	100%	87.2%
Complete vs. incomplete caries excavation	Number	20	6	8	13	2	6	4	59
	%	95.2%	100%	40%	65%	33.3%	66.7%	100%	68.6%
Size of the pulpal exposure	Number	20	6	13	10	5	7	3	64
	%	95.2%	100%	65%	50%	83.3%	77.8%	75%	74.4%
Ability to control bleeding	Number	20	6	19	18	5	9	4	81
	%	95.2%	100%	95%	90%	83.3%	100%	100%	94.2%
Post-operative restorative plan	Number	17	6	10	19	1	6	4	63
	%	80.9%	100%	50%	95%	16.6%	66.7%	100%	73.2%

In the evaluation of deep carious lesions, most participants (46.5%) do not consider age as a determining factor for selecting RCT over VPT. Meanwhile, 22.1% of participants would opt for RCT over VPT for patients aged 50 or older, 12.8% would choose RCT for both the 35-49 and 12-35 age groups, and only 5.8% would prefer RCT for patients younger than 12 years.

In terms of cost-efficiency, most participants (78%) selected VPT as a more cost-efficient treatment compared to RCT. Specifically, all the pedo faculty and pedo PG participants, as well as the majority of resto faculty (90.5%), endo faculty (75%), and endo PG (70%) participants, chose VPT as the more cost-efficient treatment option. Half of the resto PG participants and one intern participant (25%) also selected VPT. 

**Figure 3:**
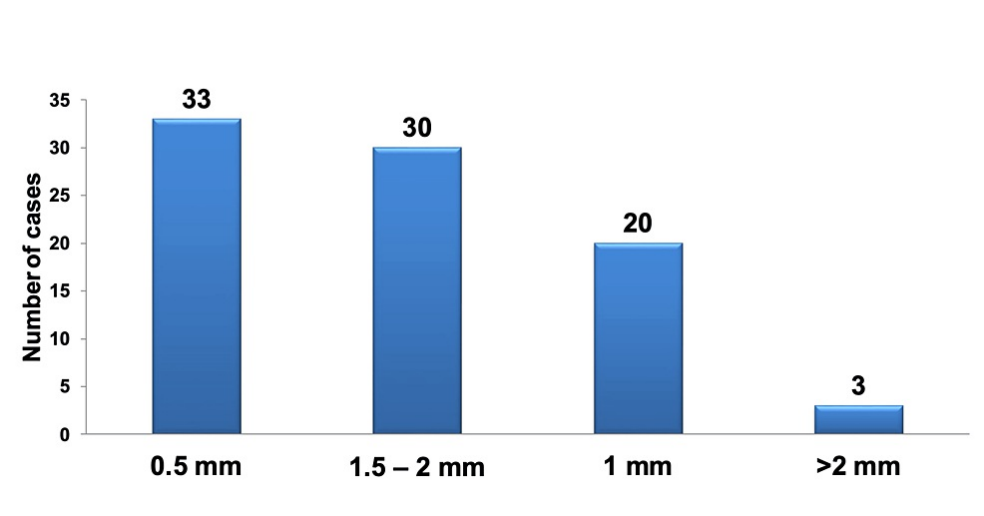
Pareto chart illustrating the minimum thickness of healthy dentin required for optimal pulp protection. The bar graph highlights that the most frequently selected minimum thicknesses were 0.5 mm, followed by the range of 1.5-2 mm, while the least preferred choice was a thickness exceeding 2 mm.

When considering the minimum thickness of healthy dentin required for optimal pulp protection, most participants tend to favor thicknesses less than 2 mm, with a pronounced preference for a minimum thickness of 0.5 mm. Selecting a minimum thickness exceeding 2 mm was notably and significantly less common than other choices, as illustrated in Figure 3 and detailed in Table 5.

**Table 5:** Multiple comparison z-test results for evaluating the minimum thickness of healthy dentin required for optimal pulp protection, regardless of specialization. Values denoted by * signify a statistically significant difference (S) with P < 0.05, while ** signifies a high level of statistical significance (HS) with P < 0.01. NS: not significant.

		Frequency	Percent	Frequency	Percent	z	P-value	
0.5 mm	1.5-2 mm	33	38.37%	30	34.88%	0.47	0.63494	P > 0.05 NS
0.5 mm	1 mm	33	38.37%	20	23.26%	2.15	0.03181*	P < 0.05 S
0.5 mm	>2 mm	33	38.37%	3	3.49%	5.62	0.00000**	P < 0.001 HS
1.5-2 mm	1 mm	30	34.88%	20	23.26%	1.68	0.09312	P > 0.05 NS
1.5-2 mm	>2 mm	30	34.88%	3	3.49%	5.23	0.00000**	P < 0.001 HS
1 mm	>2 mm	20	23.26%	3	3.49%	3.81	0.00014**	P < 0.001 HS

There were no statistically significant differences in the selections made by the different specializations regarding each minimum thickness.

Decision-making data

According to the participants, the most used material for VPT is MTA, accounting for 60.4% of the selections. Ca(OH)_2_ is the second most used material, chosen by 16.3% of the participants, followed by Biodentine at 12.8%. A small percentage of participants (10.5%) reported using other materials, with most of them using Bioceramics. It is worth noting that one participant from the endodontic faculty mentioned using any material depending on its availability (Table 6). When comparing the use of materials among different groups, the endodontic faculty and PGs reported using Biodentine more frequently than Ca(OH)_2_.

**Table 6:** Most used VPT material. The number and percentage (%) of the most used material for VPT. Resto: restorative dentistry; PG: postgraduate; Endo: endodontics; Pedo: pedodontics; VPT: vital pulp therapy; MTA: mineral trioxide aggregate.

		Resto faculty (n = 21)	Resto PG (n = 6)	Endo faculty (n = 20)	Endo PG (n = 20)	Pedo faculty (n = 6)	Pedo PG (n = 9)	Intern (n = 4)	Total (N = 86)
Ca(OH)_2_	Number	5	1	1	3	2	2	0	14
	%	23.8%	16.7%	5%	15%	33.3%	22.2%	0%	16.3%
MTA	Number	15	4	12	7	3	7	4	52
	%	71.4%	66.6%	60%	35%	50%	77.8%	100%	60.4%
Biodentine	Number	1	1	3	5	1	0	0	11
	%	4.8%	16.7%	15%	25%	16.7%	0%	0%	12.8%
Other	Number	0	0	4	5	0	0	0	9
	%	0%	0%	20%	25%	0%	0%	0%	10.5%

**Figure 4:**
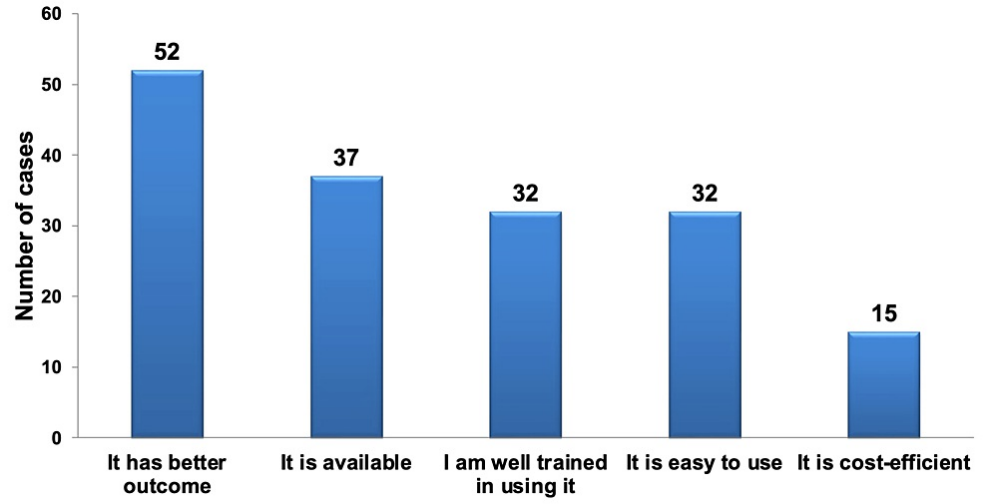
Pareto chart illustrating the rationale behind selecting the preferred material for VPT, regardless of specialization. The bar graph highlights that the most frequently cited factor was the expectation of better treatment outcomes, followed by material availability, while the least frequently considered factor was the cost-efficiency of the materials. VPT: vital pulp therapy.

Regarding the rationale behind the choice of material for VPT, it was found that the most prevalent reason across the participants was the better outcome. This was closely followed by material availability, and this difference in selection was found to be statistically significant (P < 0.05). Training level and ease of use showed the same selection frequency, ranking just below material availability, and did not exhibit a statistically significant difference. On the other hand, cost efficiency was the least frequently chosen factor, and this difference was highly statistically significant in comparison to all other factors (P < 0.01), as depicted in Figure 4 and Table 7.

**Table 7:** Multiple comparison z-test results for the rationale behind selecting the preferred material for VPT, regardless of specialization. Values denoted by * signify a statistically significant difference (S) with P < 0.05, while ** signifies a high level of statistical significance (HS) with P < 0.01. VPT: vital pulp therapy; NS: not significant.

		Frequency	Percent	Frequency	Percent	z	P Value	
It has better outcome	It is available	52	60.47%	37	43.02%	2.29	0.02209*	P < 0.05 S
It has better outcome	I am well trained in using it	52	60.47%	32	37.21%	3.05	0.00228**	P < 0.01 HS
It has better outcome	It is easy to use	52	60.47%	32	37.21%	3.05	0.00228**	P < 0.01 HS
It has better outcome	It is cost-efficient	52	60.47%	15	17.44%	5.79	0.00000**	P < 0.001 HS
It is available	I am well trained in using it	37	43.02%	32	37.21%	0.78	0.43666	P > 0.05 NS
It is available	It is easy to use	37	43.02%	32	37.21%	0.78	0.43666	P > 0.05 NS
It is available	It is cost-efficient	37	43.02%	15	17.44%	3.65	0.00026**	P < 0.01 HS
I am well trained in using it	It is easy to use	32	37.21%	32	37.21%	EQUAL VALUES		
I am well trained in using it	It is cost-efficient	32	37.21%	15	17.44%	2.91	0.00363**	P < 0.01 HS
It is easy to use	It is cost-efficient	32	37.21%	15	17.44%	2.91	0.00363**	P < 0.01 HS

Additionally, a small percentage (8.1%) of participants mentioned various other factors that influenced their material selection. These factors included comparability to Biodentine, being a gold standard material, evidence-based sealing ability, absence of discoloration, extensive clinical testing, ability to induce healing, a long history of clinical success, and high regenerative potential.

When examining the reasons guiding material selection for VPT across different specialties, there were no statistically significant differences observed in the choices related to training level, the expectation of better treatment outcomes, or cost-efficiency factors among all the specialties. However, there were statistically significant disparities between specialties concerning the factors of ease of use and material availability. For the ease-of-use factor, pedo PGs were the least likely to prioritize this factor, and this difference was highly statistically significant when compared to endo PGs alone (P < 0.01, as determined by Fisher exact probability).

Regarding the material availability factor, endo PGs tended to give this factor less importance, and this distinction was statistically significant (P < 0.05) when compared to resto PGs, highly significant (P < 0.01, as determined by Fisher Exact probability) when compared to endo faculty, and not statistically significant when compared to resto faculty. Table 8 provides details regarding the number and percentage of reasons cited for selecting the most frequently used material for VPT within each specialty.

**Table 8:** The reason for VPT material selection. The number and percentage (%) of the reason(s) to use the selected most used material for VPT. Resto: restorative dentistry; PG: postgraduate; Endo: endodontics; Pedo: pedodontics; VPT: vital pulp therapy.

		Resto faculty (n = 21)	Resto PG (n = 6)	Endo faculty (n = 20)	Endo PG (n = 20)	Pedo faculty (n = 6)	Pedo PG (n = 9)	Intern (n = 4)	Total (N = 86)
I am well trained in using it	Number	5	2	6	12	4	2	1	32
	%	23.8%	33.3%	30%	60%	66.7%	22.2%	25%	37.2%
It is easy to use	Number	5	3	5	14	3	1	1	32
	%	23.8%	50%	25%	70%	50%	11.1%	25%	37.2%
It has better outcome	Number	12	5	10	11	5	6	3	52
	%	57.1%	83.3%	50%	55%	83.3%	66.7%	75%	60.5%
It is cost-efficient	Number	2	0	1	4	4	2	2	15
	%	9.5%	0%	5%	20%	66.7%	22.2%	50%	17.4%
It is available	Number	8	4	13	3	3	3	3	37
	%	38.1%	66.7%	65%	15%	50%	33.3%	75%	43%
Other	Number	3	0	2	0	0	1	1	7
	%	14.3%	0%	10%	0%	0%	11.1%	25%	8.1%

**Figure 5:**
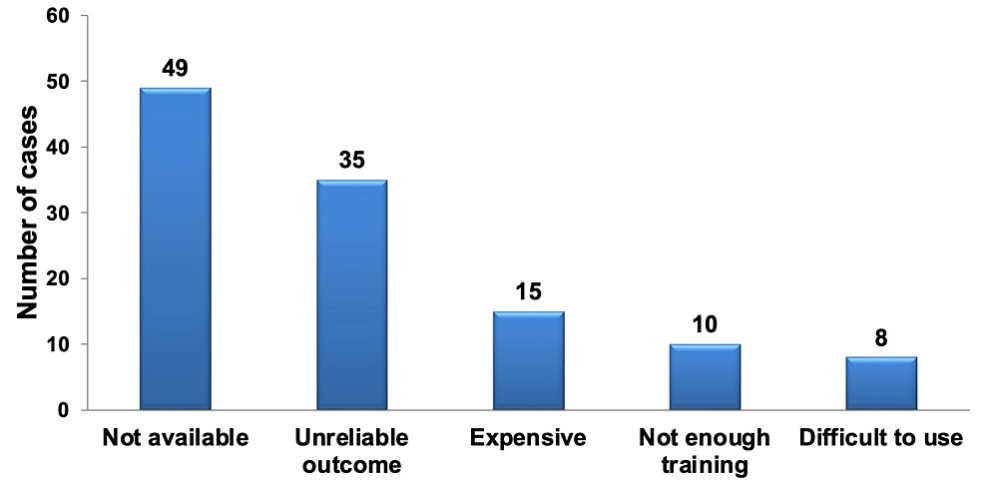
Pareto chart illustrating the rationale behind not selecting the listed materials for VPT, regardless of specialization. The bar graph highlights that the most frequently cited factor was the unavailability of the outcome of the material, followed by concerns about the material's reliability. The least commonly chosen factor was the difficulty in using the material. VPT: vital pulp therapy.

The main reason for participants, regardless of their specialization, not opting for VPT material was its unavailability. This reason was statistically significantly more common than the next factor (P < 0.05), which was the perceived unreliable outcome of the material. Both factors were highly statistically significant (P < 0.001) when compared to the other factors, as shown in Figure 5. This finding aligns with the earlier responses and underscores the credibility of the provided answers.

Among the 35 participants who selected unreliable outcome as a reason, 82.9% of them were using MTA, 11.4% were using Biodentine, 5.7% were using Bioceramics, and none of them were using Ca(OH)_2_. Some participants (11.6%) mentioned other reasons for not selecting a material for VPT, including factors such as lower efficiency, worse prognosis, long setting time, personal preference, discoloration, and high failure rate associated with the other materials.

When examining the reasons for not selecting a material for VPT among different specialties, it was observed that there were no statistically significant differences between the specialties when it came to reasons such as insufficient training, difficulty of use, unreliability of outcomes, and material unavailability. However, there was a highly significant difference (P < 0.01) in the case of material cost, where the endo and pedo faculties exhibited distinct preferences. Specifically, the endo faculty did not consider material cost as a factor in their decision not to use VPT material. Details regarding the number and percentage of selections for all specialty groups are presented in Table 9.

**Table 9:** The reason for not using the other material. The number and percentage (%) of the reason(s) selected for not using the alternative material(s) for VPT. Resto: restorative dentistry; PG: postgraduate; Endo: endodontics; Pedo: pedodontics; VPT: vital pulp therapy.

		Resto faculty (n = 21)	Resto PG (n = 6)	Endo faculty (n = 20)	Endo PG (n = 20)	Pedo faculty (n = 6)	Pedo PG (n = 9)	Intern (n = 4)	Total (N = 86)
Not enough training	Number	2	0	0	4	2	1	1	10
	%	9.5%	0%	0%	20%	33.3%	11.1%	24%	11.6%
Difficult to use	Number	1	0	3	4	0	0	0	8
	%	4.8%	0%	15%	20%	0%	0%	0%	9.3%
Unreliable outcome	Number	9	2	6	8	3	4	3	35
	%	42.9%	33.3%	30%	40%	50%	44.4%	75%	40.7%
Expensive	Number	3	1	0	4	4	2	1	15
	%	14.3%	16.7%	0%	20%	66.7%	22.2%	25%	17.4%
Not available	Number	11	3	12	10	4	6	3	49
	%	52.4%	50%	60%	50%	66.7%	66.7%	75%	57%
Other	Number	2	2	4	1	0	0	1	10
	%	9.5%	33.3%	20%	5%	0%	0%	25%	11.6%

Most participants reported using MTA and/or Biodentine for one to three years. This trend was particularly prominent among the PG students and interns, reflecting their recent clinical experience. Faculty members, on the other hand, had a higher percentage of four to six years of clinical experience using MTA and/or Biodentine. Among the faculty members, the endo faculty had the highest percentage of more than 10 years of experience with these materials, accounting for 30% of their responses.

**Figure 6:**
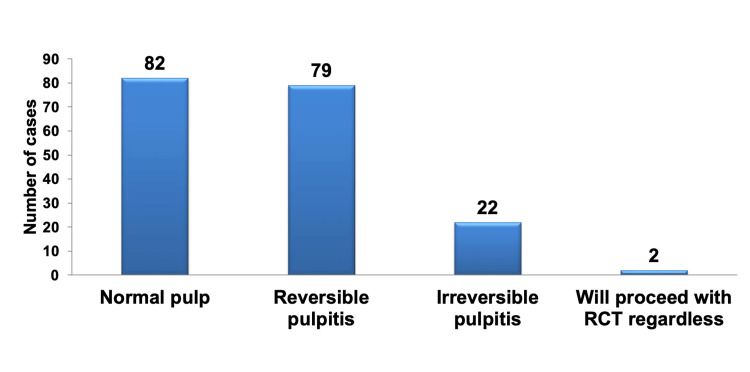
Pareto chart illustrating the pre-operative vitality test results as criteria for choosing VPT over RCT, regardless of specialization. The bar graph highlights that the most frequently selected factors were normal pulp, followed by reversible pulpitis, while the least preferred option was to proceed with RCT regardless of pre-operative vitality test results. VPT: vital pulp therapy; RCT: root canal treatment.

When opting for VPT over RCT based on pre-operative vitality test results, it was evident that most participants favored normal pulp, with reversible pulpitis coming in second. Significantly, there was no statistically significant difference in the preference between these two outcomes, and both were highly statistically significant (P < 0.01) compared to irreversible pulpitis and the decision to proceed with RCT irrespective of the pre-operative test results, as depicted in Figure 6.

There were no statistically significant differences observed among the specialties in selecting normal pulp and reversible pulpitis as the pre-operative vitality test results to determine VPT over RCT. However, when it came to choosing irreversible pulpitis, the endo PGs were the specialty with the highest preference (65%) in choosing teeth with pre-operative vitality test of irreversible pulpitis for VPT, which was statistically significant compared to the other specialties (P < 0.05), except for the endo faculty, who ranked second with a choice of 35%. Only 10% of the endo PG specialty opted for proceeding with RCT regardless of the results, and no other specialty made this selection. The total numbers and percentages for the pre-operative vitality test results indicating a preference for VPT over RCT, as well as the breakdown for each group, are presented in Table 10.

**Table 10:** Consideration of pre-operative vitality test results. The number and percentage (%) of the pre-operative vitality test results with which the participants would consider VPT over RCT. Resto: restorative dentistry; PG: postgraduate; Endo: endodontics; Pedo: pedodontics; VPT: vital pulp therapy; RCT: root canal treatment.

		Resto faculty (n = 21)	Resto PG (n = 6)	Endo faculty (n = 20)	Endo PG (n = 20)	Pedo faculty (n = 6)	Pedo PG (n = 9)	Intern (n = 4)	Total (N = 86)
Normal pulp	Number	18	6	18	13	6	8	3	82
	%	85.7%	100%	90%	65%	100%	88.9%	75%	95.3%
Reversible pulpitis	Number	19	6	19	18	5	9	3	79
	%	90.5%	100%	95%	90%	83.3%	100%	75%	91.9%
Irreversible pulpitis	Number	1	0	7	13	0	1	0	22
	%	4.8%	0%	35%	65%	0%	11.1%	0%	25.6%
Will proceed with RCT regardless	Number	0	0	0	2	0	0	0	2
	%	0%	0%	0%	10%	0%	0%	0%	2.3%

Management data

**Figure 7:**
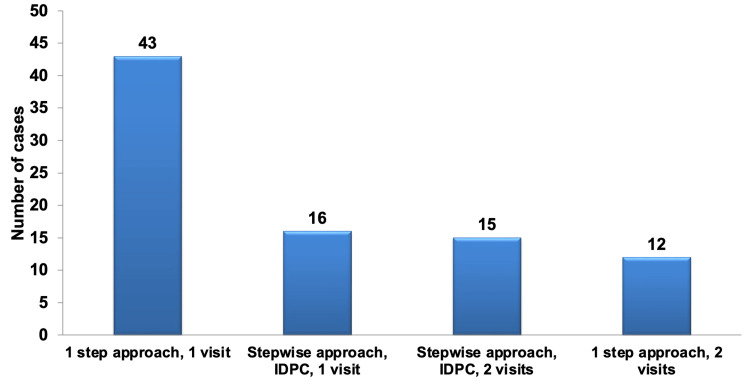
Pareto chart representing the management of deep carious lesions, regardless of specialization. The bar graph highlights that the most frequently selected approach was the one-step procedure in a single visit, while the least preferred option was the one-step approach spanning two visits. IDPC: indirect pulp capping.

In the management of deep carious lesions near the pulp, it was notable that half of the participants favored a one-step approach, involving the complete removal of the carious lesion followed by applying a base and a definitive restoration all in one visit (50%). This choice was highly statistically significant and was preferred to each of the alternative approaches, including the stepwise approach with incomplete removal of the carious lesion, indirect pulp capping (IDPC), and definitive restoration in one visit (18.6%); the stepwise approach with IDPC, temporization, and subsequent definitive restoration in another visit (17.44%); and the one-step approach with applying a base and temporary restoration, followed by definitive restoration in another visit (13.95%). These alternatives were chosen in nearly equal proportions, as illustrated in Figure 7.

Regarding the most preferred management approach, a statistically significant difference was observed, with pedo PGs abstaining from selecting the one-step approach altogether. This difference was evident when comparing pedo PGs to all other specialties, except for the interns. No statistically significant differences were found between the specialties for the other management approaches. Table 11 presents the total number and percentage of selections for each management approach when dealing with deep carious lesions near the pulp within each specialty.

**Table 11:** Treatment approach in deep carious lesions. The number and percentage (%) of the treatment approach in deep carious lesions with proximity to the pulp. Resto: restorative dentistry; PG: postgraduate; Endo: endodontics; Pedo: pedodontics; IDPC: indirect pulp capping.

		Resto faculty (n = 21)	Resto PG (n = 6)	Endo faculty (n = 20)	Endo PG (n = 20)	Pedo faculty (n = 6)	Pedo PG (n = 9)	Intern (n = 4)	Total (N = 86)
One-step approach, two visits	Number	1	1	7	2	0	0	1	12
	%	4.8%	16.7%	35%	10%	0%	0%	25%	14%
One-step approach, one visit	Number	10	3	12	16	1	0	1	43
	%	47.6%	50%	60%	80%	16.7%	0%	25%	50%
Stepwise approach, IDPC, two visits	Number	6	0	1	2	1	3	2	15
	%	28.6%	0%	5%	10%	16.7%	33.3%	50%	17.4%
Stepwise approach, IDPC, one visit	Number	4	2	0	0	4	6	0	16
	%	19%	33.3%	0%	0%	66.7%	66.7%	0%	18.6%

**Figure 8:**
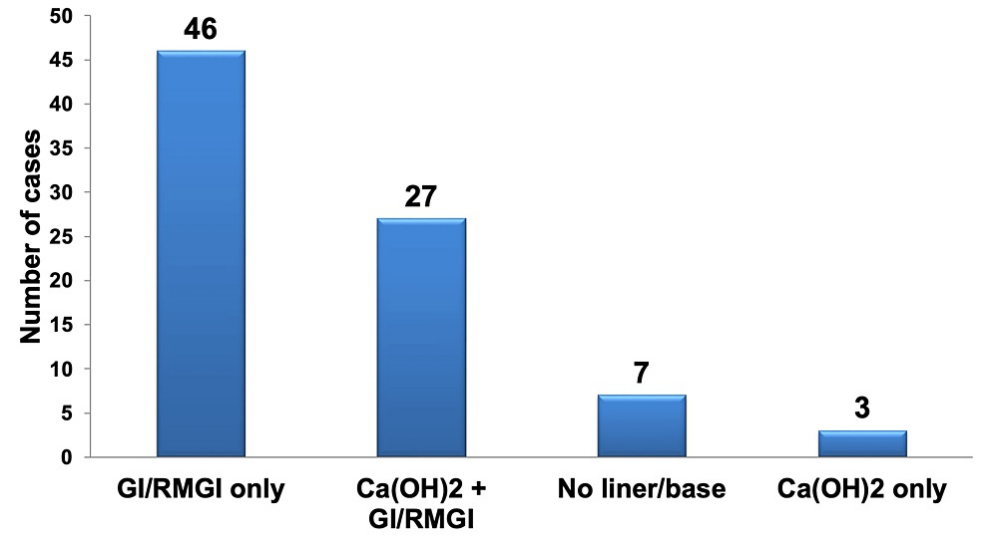
Pareto chart depicting the management of deep carious lesions without pulpal exposure after thorough caries excavation, for cavities that will undergo resin composite restoration, regardless of specialization. The bar graph highlights that the most frequently selected materials were GI or RMGI, while the least preferred material was Ca(OH)_2_. GI: glass ionomer; RMGI: resin-modified glass ionomer.

In the management of deep carious lesions without pulpal exposure, after complete caries excavation for cavities to be restored with resin composite material, the most frequently selected materials for pulp protection were GI or RMGI base only (53.49%). This choice was highly statistically significant (P < 0.01) when compared to the second-most preferred materials, which were Ca(OH)_2_ liner and GI or RMGI base (31.40%). Both of these preceding choices were highly statistically significant (P < 0.001) compared to opting for no liner or base (8.14%), using a Ca(OH)_2_ liner only (3.49%), or selecting other materials (3.48%), as illustrated in Figure 8.

When comparing the selections made by different specialties, it was observed that there were no statistically significant differences among the groups in choosing GI/RMGI base only, no liner or base, and Ca(OH)_2_ liner only (P > 0.05). However, there was a statistically significant difference among the specialties in selecting the use of both Ca(OH)_2_ liner and GI or RMGI base. In this case, the resto and endo faculty members chose this approach significantly less frequently (P < 0.05) than the interns.

Among all the participants, only three endo faculty members (3.5%) opted for other materials as their management approach. They mentioned the use of materials such as MTA or bioceramic materials, with some using GI base. One faculty member mentioned employing temporary restorations and referring the patient for a restorative decision. Table 12 presents the total number and percentage of selections for pulpal protection in the management of deep carious lesions without pulpal exposure, after complete caries excavation, for cavities to be restored with resin composite, categorized by each specialty.

**Table 12:** Management of deep carious lesions without pulp exposure. The number and percentage (%) of the management in cavities with proximity to the pulp without pulp exposure that will be restored with resin composite material. Resto: restorative dentistry; PG: postgraduate; Endo: endodontics; Pedo: pedodontics; GI: glass ionomer; RMGI: resin-modified glass ionomer.

		Resto faculty (n = 21)	Resto PG (n = 6)	Endo faculty (n = 20)	Endo PG (n = 20)	Pedo faculty (n = 6)	Pedo PG (n = 9)	Intern (n = 4)	Total (N = 86)
Ca(OH)_2_ only	Number	0	1	2	0	0	0	0	3
	%	0%	16.7%	10%	0%	0%	0%	0%	3.5%
Ca(OH)_2_ + GI/RMGI	Number	3	2	3	10	2	4	3	27
	%	14.3%	33.3%	15%	50%	33.3%	44.4%	75%	31.4%
GI/RMGI only	Number	14	2	11	9	4	5	1	46
	%	66.7%	33.3%	55%	45%	66.7%	55.6%	25%	53.5%
No liner/base	Number	4	1	1	1	0	0	0	7
	%	19%	16.7%	5%	5%	0%	0%	0%	8.1%
Other	Number	0	0	3	0	0	0	0	3
	%	0%	0%	15%	0%	0%	0%	0%	3.5%

**Figure 9:**
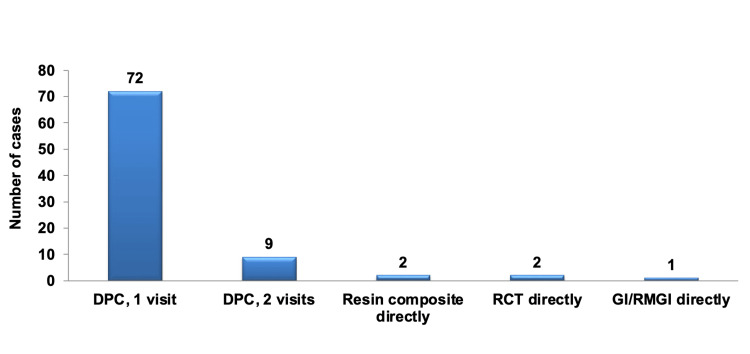
Pareto chart illustrating the management of deep carious lesions with pulpal exposure after complete caries excavation, adequate isolation, controlled bleeding, and no contamination, regardless of specialization. The bar graph highlights that the most selected approach by most participants was DPC in a single visit. DPC: direct pulp capping; RCT: root canal treatment.

Conversely, when considering the management and treatment protocol steps for cases involving deep carious lesions with pulpal exposure following complete caries excavation, adequate isolation, controlled bleeding, and no contamination, the predominant and most favored approach chosen by participants was to place a direct pulp capping (DPC) material, a base, and a definitive restoration all in the same visit (one-visit), accounting for 83.72% of selections. This choice was highly statistically significant when compared to all other management options (P < 0.001), including placing a DPC material and temporary filling followed by a definitive restoration at a subsequent visit (two-visits) (10.47%), using a well-sealed adhesive and resin composite directly (2.33%), proceeding with RCT (2.33%), and employing a long-term temporary filling (GI or RMGI) (1.16%). The remaining management approaches did not exhibit statistically significant differences in their selection from one another, as depicted in Figure 9.

There were no statistically significant differences observed in the selection of any of the management steps among the different specialties. Table 13 presents the total number and percentage of selections for the management protocol in cases of deep carious lesions with pulpal exposure, categorized by each specialty.

**Table 13:** Management of deep carious lesions with pulp exposure. The number and percentage (%) of the management in cavities with pulp exposure after complete caries excavation, sufficient isolation, controlled bleeding, and no contamination. Resto: restorative dentistry; PG: postgraduate; Endo: endodontics; Pedo: pedodontics; DPC: direct pulp capping; RCT: root canal treatment; GI: glass ionomer; RMGI: resin-modified glass ionomer.

		Resto faculty (n = 21)	Resto PG (n = 6)	Endo faculty (n = 20)	Endo PG (n = 20)	Pedo faculty (n = 6)	Pedo PG (n = 9)	Intern (n = 4)	Total (N = 86)
DPC, 2 visits	Number	1	1	3	2	1	0	1	9
	%	4.8%	16.7%	15%	10%	16.7%	0%	25%	10.5%
DPC, 1 visit	Number	18	5	15	18	5	8	3	72
	%	85.7%	83.3%	75%	90%	83.3%	88.9%	75%	80%
Resin composite directly	Number	1	0	0	0	0	1	0	2
	%	4.8%	0%	0%	0%	0%	11.1%	0%	2.3%
RCT directly	Number	0	0	2	0	0	0	0	2
	%	0%	0%	10%	0%	0%	0%	0%	2.3%
GI/RMGI directly	Number	1	0	0	0	0	0	0	1
	%	4.8%	0%	0%	0%	0%	0%	0%	1.2%

When managing cases where the pulp remains vital but has experienced iatrogenic pulpal exposure, most participants (62.79%) opted for MTA as the material of choice for direct pulp protection, considering it to produce the best biological outcomes such as pulpal repair and dentin bridge formation. This selection was highly statistically significant (P < 0.001) when compared to the second most favored material, Biodentine (29.07%). Both MTA and Biodentine were chosen more frequently and were highly statistically significant (P < 0.001) compared to all the other options available.

**Figure 10:**
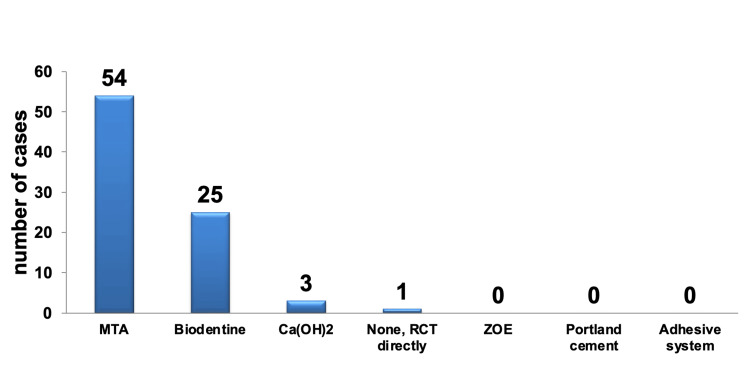
Pareto chart representing the selection of materials for DPC to achieve optimal biological outcomes, without consideration of specialization. The bar graph highlights that the most frequently chosen materials were MTA, followed by Biodentine. DPC: direct pulp capping; MTA: mineral trioxide aggregate; RCT: root canal treatment; ZOE: zinc oxide and eugenol.

In contrast, the remaining options, including the use of Ca(OH)_2_ (3.49%), other materials (3.49%), and immediate initiation of RCT (1.16%), were chosen less frequently and did not exhibit statistically significant differences from each other. Moreover, these less preferred options were also not statistically significant when compared to the options that were not chosen at all, including zinc oxide and eugenol (ZOE), Portland cement, and adhesive system materials, as depicted in Figure 10.

Only MTA and Biodentine had a sufficient amount of data to conduct a statistical analysis between the different specialties. Regarding the selection of MTA, there was no statistically significant difference observed among the specialties (P > 0.05).

However, when it came to the selection of Biodentine, a statistically significant difference was identified between pedo PGs and both resto and endo PGs. In this comparison, pedo PGs tended to choose Biodentine to a lesser extent (P < 0.05).

A small percentage (3.49%) of participants who opted for other materials mentioned using Bioceramics for DPC, and all of them belonged to the endodontic specialty, including endo PGs and endo faculty members. Table 14 provides details on the total number and percentage of selections for the choice of DPC material to achieve the best biological outcomes, categorized by each specialty.

**Table 14:** Preferred DPC material. The number and percentage (%) of the DPC material of choice to produce the best biological results (pulp repair, and dentin bridge formation). Resto: restorative dentistry; PG: postgraduate; Endo: endodontics; Pedo: pedodontics; DPC: direct pulp capping; MTA: mineral trioxide aggregate; RCT: root canal treatment; ZOE: zinc oxide and eugenol.

		Resto faculty (n = 21)	Resto PG (n = 6)	Endo faculty (n = 20)	Endo PG (n = 20)	Pedo faculty (n = 6)	Pedo PG (n = 9)	Intern (n = 4)	Total (N = 86)
ZOE	Number	0	0	0	0	0	0	0	0
	%	0%	0%	0%	0%	0%	0%	0%	0%
Portland cement	Number	0	0	0	0	0	0	0	0
	%	0%	0%	0%	0%	0%	0%	0%	0%
Ca(OH)_2_	Number	0	0	0	1	0	2	0	3
	%	0%	0%	0%	5%	0%	22.2%	0%	3.5%
MTA	Number	16	3	11	10	4	7	3	54
	%	76.2%	50%	55%	50%	66.7%	77.8%	75%	62.8%
Biodentine	Number	5	3	6	8	2	0	1	25
	%	23.8%	50%	30%	40%	33.3%	0%	25%	29%
Adhesive system	Number	0	0	0	0	0	0	0	0
	%	0%	0%	0%	0%	0%	0%	0%	0%
None, RCT directly	Number	0	0	1	0	0	0	0	1
	%	0%	0%	5%	0%	0%	0%	0%	1.1%
Other	Number	0	0	2	1	0	0	0	3
	%	0%	0%	10%	5%	0%	0%	0%	3.5%

## Discussion

The survey's sample size is 86 participants, which falls short of the calculated sample size of 108, resulting in a moderate bias. This discrepancy may have a moderate impact on the survey's representativeness and the reliability of the results. Traditionally, VPT has primarily focused on preserving the radicular pulp in immature adult teeth to ensure the completion of root formation (apexogenesis). However, the scope of VPT has expanded in recent years, offering practitioners alternative treatment options for mature teeth that were previously believed to have irreversibly inflamed pulps. Most participants in the survey considered pre-operative vitality test results as the most crucial factor when assessing deep carious lesions for VPT or RCT. This indicates that participants prioritize evaluating the vitality of the tooth before deciding on the treatment approach. Pre-operative diagnosis was an area of conflict among participants from different disciplines. While participants from restorative and pediatric departments leaned towards RCT, 65% of endo PGs and 35% of endo faculty members opted for VPT in these cases. This discrepancy potentially reflects the expertise and confidence of endo specialists in managing such cases. It also reflects the revised recommendation of the AAE as presented in the positional statement released in 2021 that recommended expansion in the use of VPT to address cases with a pre-treatment diagnosis of irreversible pulpitis [19]. A systematic review focused on teeth with signs and symptoms indicative of irreversible pulpitis reported an average success rate of 97.4% clinically and 95.4% radiographically for coronal pulpotomy at a 12-month follow-up [20].

The age of the patient and the remaining tooth structure were also significant factors in the assessments of survey participants. In this survey, it was found that interns tended to prioritize socio-economic status more than endo PGs as a factor in decision-making. On the other hand, when it comes to the oral hygiene factor, the endo faculty tended to consider this factor more important compared to the resto faculty. Other factors, such as access to treatment/patient compliance, were considered but to a slightly lesser extent. The level of isolation during caries excavation, the size of pulpal exposure, the post-operative restorative plan, and the completeness of caries excavation were also identified as important factors indicating a comprehensive approach to treatment planning and management. Studies that investigated the outcome of VPT and influencing factors, such as age and sex of the patient, type of tooth, and jaw, reported those not found to be significant in the outcome of VPT.

In cases of pulpal exposure, complete removal of caries is crucial to eliminate infected tissues and enable the visualization of pulp tissue conditions under magnification [21]. Residual caries hinder the accurate assessment of pulpal inflammation and potential necrotic areas. Thus, effective management of vital pulp tissue necessitates the complete removal of demineralized enamel and infected dentin. Instead of avoiding pulp exposure, the clinician should prioritize the thorough removal of demineralized infected dentin to enhance the prospects of successful pulpal repair [22].

In this survey, when assessing pulpal exposure following the excavation of deep carious lesions, participants highlighted the ability to control bleeding as the most crucial factor. This emphasizes the importance of achieving hemostasis during treatment. To facilitate the clinical assessment of inflammatory levels and identify potential necrotic tissues that need to be removed prior to the application of a suitable biomaterial, it is essential to achieve hemostasis. Hemostasis of the pulp tissue is commonly achieved by bathing the resected pulp tissue in sodium hypochlorite for a duration of five to 10 minutes, although recommended durations may differ. This can be achieved either through direct passive irrigation or by placing a sodium hypochlorite-soaked cotton pellet. These techniques aim to control hemorrhage and create a suitable environment for subsequent treatment procedures [19].

In terms of cost-efficiency, most participants (78%) selected VPT as a more cost-efficient treatment compared to RCT. In a study by Schwendicke and Stolpe, they investigated the long-term cost-effectiveness of VPT versus RCT in the treatment of carious permanent teeth. The reported VPT was more cost-effective in younger patients and for occlusal exposure sites, whereas RCT was more effective in older patients or teeth with proximal exposures [23]. 

When evaluating the minimum thickness of healthy dentin required for optimal pulp protection, most participants tend to favor thicknesses less than 2 mm, with a pronounced preference for a minimum thickness of 0.5 mm. It has been reported that 0.5 mm of remaining dentin thickness or greater is necessary to avoid evidence of pulp injury following restoration [24].

In a prospective study conducted by Taha and Khazali [25], the outcome of VPT was investigated in teeth diagnosed with irreversible pulpitis. A partial pulpotomy was performed on 50 permanent molars, and the teeth were randomly assigned to receive either white MTA or Ca(OH)_2_ as the pulpotomy agent. At the two-year follow-up, the success rate in the MTA group was nearly twice that of the Ca(OH)_2_ group [25]. In another study exploring the use of bioceramic material as a pulp capping agent for teeth with irreversible pulpitis, the overall clinical and radiographic success rate of VPT was reported to be 90.5% over a follow-up period of one year or more. Comparing the outcomes of VPT for symptomatic teeth using two different types of cement, ProRoot MTA and Biodentine, a similar success rate was observed. However, Biodentine exhibited significantly less discoloration compared to ProRoot MTA [26]. In the current survey, MTA was the most selected material for VPT followed by Ca(OH)_2_ and Biodentine among all participants from the different specialties. 20% of Endo faculty and 25% of Endo PG reported using Bioceramics such as BC Putty (Brasseler, Savannah, USA) for VPT.

The selection of a biomaterial for pulp capping should be based on available evidence, considering patient-centered outcomes, reliable formation of mineralized tissue, and preservation of pulp vitality. Numerous studies have demonstrated that Ca(OH)_2_ is not an ideal material for pulp capping. It lacks adhesion to dentin and undergoes dissolution over time [27]. Additionally, the hard tissue bridge formed beneath Ca(OH)_2_ often exhibits imperfections and tunnel defects, which may allow bacterial microleakage [28]. In contrast, a histological study using animal models has shown that when MTA is placed against pulp tissue, the resulting bridge is complete, composed of tubular structures, and free from tunnels or imperfections [29]. In addition, the alkalinity of MTA, along with its release of calcium ions and maintenance of a sustained pH of 12.5, is likely responsible for preventing further microbial growth from residual microorganisms that may remain after the removal of carious tissue. Calcium silicate cements (CSCs) have emerged as a promising option for VPT procedures [30]. CSCs encompass various materials such as tricalcium silicates, dicalcium silicates, hydraulic CSC, and "bioceramics." Clinical outcomes have consistently demonstrated success rates comparable to those of MTA. The newer generations of CSCs exhibit improved setting times [31]. When MTA and other CSCs are used for VPT in permanent teeth with symptomatic or asymptomatic irreversible pulpitis, success rates range from 85% to 100% at one to two years.

A randomized clinical trial was conducted to compare the efficacy of MTA with a conventional Ca(OH)_2_ liner (Dycal) as DPC materials in adult molars with carious pulpal exposure. After a follow-up period of 36 months, the success rate in the MTA group was determined to be 85%, whereas it was 52% in the Ca(OH)_2_ group [32]. A retrospective study was conducted to examine the outcomes of DPC using either MTA or Ca(OH)_2_ paste. In the MTA group, 78% of cases were classified as successful and 22% as failures. In the Ca(OH)_2_ group, 60% were classified as successful and 40% as failures. When examining the rationale for material selection for VPT, it was found that the most prevalent reason across all participants was the better outcome. This was closely followed by material availability. Training level, ease of use, and cost efficiency were not major factors influencing material selection across all specialties.

In the current survey, all participants selected a treatment option where the definitive restoration is placed at the first visit when treating a tooth with a deep carious lesion or when a pathological or iatrogenic exposure is encountered if the tooth is isolated and bleeding is controlled. This is in agreement with previously reported findings that immediate and definitive restoration of teeth following direct pulp capping was found to positively impact treatment outcomes, regardless of the type of capping material used [33].

Recent clinical studies have reported the utilization of novel materials for VPT that would allow a single-visit treatment option. Asgary et al. investigated the efficacy of calcium-enriched mixture cement as a base for a VPT in managing deep caries in mature permanent molars [21]. This study included teeth exhibiting clinical signs of irreversible pulpitis and the presence of apical periodontitis, with a total of 302 teeth randomly assigned to four VPT techniques: IDPC, DPC, partial pulpotomy (PP), and full pulpotomy (FP). The study evaluated the success rates of these VPT techniques at three and 12 months. Remarkably, the outcomes demonstrated comparable success rates across all four techniques, with IDPC (98.7%), DPC (98.4%), PP (98.4%), and FP (93.5%) exhibiting favorable success rates at both time intervals [21].

When comparing the responses of participants in the current study to those in previous investigations concerning treatment preferences for permanent teeth with carious pulp exposure among general dentists and endodontists working in Thai public hospitals, several consistent patterns emerge. Decision-making was notably influenced by clinical symptoms. In this study, VPT, particularly direct pulp capping, emerged as the preferred treatment choice among all participants. In cases of teeth with clinical signs and symptoms of irreversible pulpitis, participants with postgraduate education preferred vital pulp therapy over apexification or RCT. Factors such as the ability to control bleeding, remaining dentin thickness, and accessibility to treatment were not investigated [34]. Comparable results were observed in a survey that examined the management of deep caries and exposed pulp among members of the Endodontic Society in Ireland and Italy. Notably, the presence of patient symptoms and the patient's age emerged as significant factors influencing the decision-making process and the choice of treatment, with implications for the level of invasiveness of treatment [16]. A study conducted in China investigated the choice of treatment procedure and material selection among dentists versus endodontists in the management of permanent teeth with deep carious lesions. Endodontists and senior respondents displayed a preference for DPC over PP or RCT in contrast to general practitioners. Additionally, they favored the use of calcium silicate materials (CSMs) for VPT as opposed to MTA or fast-setting Ca(OH)_2 _[35].

During the placement of pulp capping material, it is advised to apply MTA or CSCs over the sites of exposure and a significant portion of the surrounding dentin to entomb any remaining microorganisms [36]. This notable shift in pulp capping strategy can enhance treatment outcomes in teeth with symptomatic or asymptomatic conditions, characterized by extensive caries and multiple exposures following caries excavation [37]. Furthermore, ensuring a cement thickness of 1.5 mm or more when applying MTA or CSC promotes effective neutralization of bacteria and reduces the likelihood of subsequent microbial challenges.

Most participants preferred performing pulp capping, placing a base, and applying the definitive restoration all in the same visit for the management of cavities near the pulp. This indicates a preference for immediate restoration and management of cavities to provide protection and minimize the risk of further complications or contamination. Previous studies have highlighted that the main cause of failure in vital pulp therapy is leakage during the healing process [38]. Barthel et al. reported that the success rate of pulp capping for carious exposures in permanent teeth was significantly influenced by placing the definitive restoration within the first two days following the exposure [10]. Furthermore, when examining the relationship between the choice of restorative material and treatment outcome, it was observed that cavities restored with GIC had significantly poorer outcomes compared to all other restorative materials such as amalgam, composite resin, ceramic, and gold [39].

The survey's limitation lies in its moderate response rate, which falls within an acceptable range but necessitates consideration of potential non-response bias. Unfortunately, we couldn't examine the pattern of non-response due to a lack of information about the segment of the population that did not participate. Possible explanations for this non-response could include time constraints, lack of interest, or the busy schedules of both faculty and students at the facility. On a positive note, the survey boasts several advantages. It features an extensive array of questions covering five major clinical aspects, involving professionals from various specialties and general practitioners across different education levels. These individuals share a common interest in addressing issues related to deep caries encounters.

Procedural determinations regarding the extent of pulp tissue preservation or removal should be guided by the operator's assessments, clinical judgment, overall treatment plan, and the oral and systemic health status of the patient. Further clinical trials are necessary to evaluate the long-term effectiveness of vital pulp therapy as well as the development of chair-side techniques that incorporate biomarkers for assessing pulpal viability.

## Conclusions

For the participants in this study, pre-operative vitality test results are the most crucial factor when assessing deep carious lesions for VPT or RCT. Most participants would consider VPT over RCT when the pre-operative vitality test results indicate normal pulp. While participants from restorative and pediatric departments leaned towards RCT in symptomatic cases, 65% of endo PGs and 35% of endo faculty members opted for VPT in symptomatic cases. The ability to control bleeding following pulpal exposure was highlighted as a central factor in the decision-making for VPT versus RCT among all participants. The most used material for VPT among all participants is MTA, followed by Ca(OH)_2_ and Biodentine. Factors such as access to treatment/patient compliance, remaining dentin thickness, and oral hygiene of the patient were of minimal significance in the selection of treatment procedures. The primary reason for selecting VPT material, as reported by the participants, was the better outcome. This was closely followed by material availability. Most participants preferred placing pulp capping, placing a base, and applying the definitive restoration all in the same visit for the management of cavities near the pulp or following pulpal exposure.

## Appendices

The survey questions related to management of deep carious lesions and VPT.

Demographic, Education, and Experience Data

What is your age range in years?

  23-29

  30-39

  40-49

  50-59

  60-90

What is your gender?

  Male

  Female

What is your current level of dental training?

  Intern

  Pedodontic postgraduate

  Endodontic postgraduate

  Restorative/operative postgraduate

  Pedodontic faculty

  Endodontic faculty

  Restorative/operative faculty

Which residency (R) level are you currently at? This question will only appear if the participant chose one of the PG options.

  R1

  R2

  R3

When was the year of your postgraduate clinical qualification? This Q will only appear for participants who chose one of the faculty options.

  Before 2000

  In 2000

  After 2000

Where did you get your PG qualification from? This Q will only appear for participants who chose one of the faculty options.

  Saudi Arabia

  Arab countries other than Saudi Arabia

  UK or other European countries

  USA

  Other

What type of clinical practice are you currently in?

  Governmental institute

  Private

  Both governmental and private

  I do not practice clinically (academic only)

What is the estimate number of VPT cases you practice per month? This Q will not appear for participants who chose that they are not practicing clinically (Academicians).

  0

  1-5

  6-10

  >10

What is/are the material(s) that you have ever used at any time for VPT (Choose all that applies) This Q will not appear for participants who chose that they are not practicing clinically (Academicians).

  Calcium hydroxide Ca(OH)2

  Mineral trioxide aggregates (MTA)

  Biodentine

  Other

Assessment Data

What are the factors that you will consider in assessing cases of deep carious lesions for either VPT or RCT before the start of the treatment? (Choose all that applies)

  Age of the patient

  Socio-economic status of the patient

  Oral hygiene of the patient

  Remaining dentin thickness over the pulp after caries excavation

  Pre-operative vitality test result

  Remaining tooth structure

  Access to treatment and/or patient compliance

What are the factors that you will consider in assessing cases of deep carious lesions for VPT or RCT following iatrogenic pulpal exposure? (Choose all that applies)

  Level of isolation during caries excavation

  Complete versus incomplete caries excavation at the time of exposure

  Size of the pulpal exposure

  Ability to control bleeding

  Post-operative restorative plan

At what age would you more likely choose RCT rather than VPT?

  <12 years

  12-35 years

  35-49 years

  >50 years

  Age is not a critical factor in my decision

Which treatment option is more cost-efficient?

  VPT

  RCT

What is the minimum thickness of sound dentin needed for the optimum protection of the pulp following VPT?

  0.5 mm

  1 mm

  1.5-2 mm

  >2 mm

Decision-making Data

Which material do you use the most for VPT?

  Ca(OH)2

  MTA

  Biodentine

  Other

What is/are the reason(s) you use the material you chose in the Q15 over the others? (Choose all that applies)

  I am well-trained in using it

  It is easy to use

  It has better outcome

  It is cost-efficient

  It is available

  Other

What is/are the reason(s) for not using the other materials listed but not chosen by you in Q15? (Choose all that applies)

  Not enough training in its/their use

  Difficult to use

  Unreliable outcome

  Expensive

  Not available

  Other

If you are using MTA and/or Biodentine for VPT, how long have you been using it/them?

  1-3 years

  4-6 years

  7-9 years

  >10 years

  I do not use MTA and Biodentine

With which pre-operative vitality test result(s) would you consider VPT over RCT? (Choose all that applies)

  Normal pulp

  Reversible pulpitis

  Irreversible pulpitis

  I will proceed with endodontic treatment regardless of the pre-operative pulpal status

Management

In cases of deep carious lesions with pulp proximity, what would be your treatment option?

  Total removal of the carious lesion (one-step approach), applying a base material, temporary restoration, then replacement by definitive restoration in another clinical visit

  Total removal of the carious lesion (one-step approach), applying a base material, and definitive restoration in the same clinical visit

  Incomplete removal of the carious dentin in the deep or of the cavity (stepwise approach), indirect pulp capping, temporary restoration, followed by definitive restoration after completing caries excavation in another clinical visit

  Incomplete removal of the carious dentin in the deep or of the cavity (stepwise approach), indirect pulp capping, followed by definitive restoration in the same clinical visit

In cases of deep carious lesions without pulpal exposure after complete caries excavation that will be restored with resin composite material, how would you proceed to protect the pulp?

  Ca(OH)2 liner only

  Ca(OH)2 liner + glass ionomer or resin-modified glass ionomer base

  Glass ionomer or resin-modified glass ionomer base only

  No need for liner or base

  Other

In cases of deep carious lesions with pulpal exposure after complete caries excavation, sufficient isolation, controlled bleeding, and no contamination. What would be the steps of your treatment protocol?

  Place pulp capping material and temporary lining (Cavit), follow-up for definitive restoration later

  Place pulp capping material, a base, and definitive restoration in the same visit

  Place well-sealed adhesive and resin composite directly

  Proceed with RCT

  Long-term temporary lining (glass ionomer or resin-modified glass ionomer)

In cases where the tooth pulp is vital and the pulp was exposed and well isolated, which material would you choose for direct protection aiming to produce the best biological results (pulp repair, with dentin bridge formation)?

  Zinc oxide with eugenol cement

  Portland cement

  Ca(OH)2 cement

  MTA

  Biodentine

  Adhesive system

  None, I would proceed with RCT

  Other
